# Organization of Lipids in the Tear Film: A Molecular-Level View

**DOI:** 10.1371/journal.pone.0092461

**Published:** 2014-03-20

**Authors:** Alicja Wizert, D. Robert Iskander, Lukasz Cwiklik

**Affiliations:** 1 Institute of Biomedical Engineering and Instrumentation, Wroclaw University of Technology, Wroclaw, Poland; 2 J. Heyrovský Institute of Physical Chemistry, Academy of Sciences of the Czech Republic, v.v.i., Prague, Czech Republic; 3 Institute of Organic Chemistry and Biochemistry, Academy of Sciences of the Czech Republic, v.v.i., Prague, Czech Republic; 4 Department of Physics, Tampere University of Technology, Tampere, Finland; Jacobs University Bremen, Germany

## Abstract

Biophysical properties of the tear film lipid layer are studied at the molecular level employing coarse grain molecular dynamics (MD) simulations with a realistic model of the human tear film. In this model, polar lipids are chosen to reflect the current knowledge on the lipidome of the tear film whereas typical Meibomian-origin lipids are included in the thick non-polar lipids subphase. Simulation conditions mimic those experienced by the real human tear film during blinks. Namely, thermodynamic equilibrium simulations at different lateral compressions are performed to model varying surface pressure, and the dynamics of the system during a blink is studied by non-equilibrium MD simulations. Polar lipids separate their non-polar counterparts from water by forming a monomolecular layer whereas the non-polar molecules establish a thick outermost lipid layer. Under lateral compression, the polar layer undulates and a sorting of polar lipids occurs. Moreover, formation of three-dimensional aggregates of polar lipids in both non-polar and water subphases is observed. We suggest that these three-dimensional structures are abundant under dynamic conditions caused by the action of eye lids and that they act as reservoirs of polar lipids, thus increasing stability of the tear film.

## Introduction

Tear film is important to the health and optics of the human eye [1], [2]. It refreshes with every blink, undergoes several phases of its kinetics, and eventually ruptures if blinking is suppressed [3]. Tear film instabilities lead to symptoms known as dry eye syndrome, one of the commonly reported eye ailments [4]. When untreated, it can lead to blindness. Dry eye is significantly more common in females than males and it is highly prevalent in contact lens wearers [5]–[9].

Although tear film has been studied for decades, there is still much to be discovered in relation to its composition and function at both macroscopic and microscopic scales. The classical textbook distinct-three-layer structure that divides tear film into lipid, aqueous and mucin layers has been extensively researched and discussed [10]–[14]. Some studies of tear film focus on its entire structure, other concentrate on its biochemical and biophysical characteristics. In many cases, the macroscopic view studies have an abridged interpretation of the underlying biochemistry while those focusing on biochemical compositions and molecular level biophysics often do not fully cover the effects their results have on tear film macroscopic scale function. The classical model of tear film formation is also debated [15]. There is no unified theory that would provide comprehensive foundations for explaining the microscopic and macroscopic behavior of the human tear film. This is attributed to the inherent complexity of the tear film and technical difficulties in measuring its characteristics in vivo [16].

Of particular interest of study is the outermost layer of tear film, often referred to as the tear film lipid layer (TFLL) [17]–[19]. Its major function is to provide an optically smooth surface over the cornea and retarding evaporation from the eye [17], [20]–[22]. With the help of macroscopic view techniques, specific patterns in the outer tear film layer were observed and related to the lipid distribution without direct lipid identification. Changes in lipid pattern, distribution, and thickness were all associated with dry eye syndrome [19], [23]. Some studies tried to distinguish two groups of lipids (polar and non-polar) to account for abnormalities encountered in keratoconjunctivitis sicca – a particular type of dry eye caused by either decreased tear production or increased tear film evaporation [24]. The tear lipocalins [25], [26] that are considered to contribute to the high non-Newtonian viscosity of the tear film, its low surface tension and tear film lipid layer kinetics were also found to be altered in dry eyes [27].

Many studies focused on lipids present in the meibomian gland [28]–[30] because, until recently, it was presumed that the tear film lipid layer is almost exclusively produced by those glands. This common view has been changed by recent studies on the composition of tear film lipids. Butovich has qualitatively compared the nonpolar lipids that are secreted from the meibomian gland to those present in aqueous tears [31]. The latter were collected from the lower tear menisci using either glass microcapillaries or Schirmer test strips. Rantamäki et al. attempted to identify the human aqueous tear fluid lipidome [32] based on a collective sample from a group of subjects. Both studies clearly showed that the meibomian glands could not be the only source of lipids in the tear film lipid layer. These results confirmed earlier chemical analyses of tear film lipids [33], indicating that previously reported macroscopic view studies of tear film are in need of revision [34]–[38].

Due to the abovementioned complexity of the tear film lipid layer and difficulties in imaging its microscopic behavior [39], [40], simulation studies utilizing the tools of molecular dynamics have been recently conducted [32], [41]–[43]. The works essentially focused on the air-water interface of a lachrymal glad fluid. In the context of the stability of the tear film lipid layer, structural and temporal properties of the interface were studied at different levels of surface pressure.

Encouraged by the results of recent molecular dynamics studies, our objective was to increase the scale of the tear-air interface and include in the study both lipids present in the tear film and those originating from the meibomian gland. In this way we aimed to study biophysical properties of a realistic tear film model at the molecular level and under conditions that physiologically occur during blinking.

## Methodology

The simulated systems contained water, polar lipids, and non-polar lipids. The lipid composition employed mimics the experimentally assessed lipidome of the tear film, as discussed in the Introduction. More specifically, the choice of polar lipids and their concentrations directly reflect the current knowledge about lipidome of the aqueous tear fluid [32], whereas two experimentally most abundant non-polar lipids were selected here to model the non-polar lipid layer [34]. The previous computational studies of tear film aimed at explaining the role of the polar to non-polar lipids ratio for the tear film stability [42], [43]. Systems with a relatively thin, about monomolecular, layer of polar lipids directly covering the water phase, and with only a minor component of non-polar lipids deposited on the polar layer were considered therein. Based on experimental studies, the non-polar sub-layer present in the TFLL is expected to be substantially thicker than the polar one, with the estimated thickness of up to 90 nm [34], [40]. Hence, to capture essential features of the whole TFLL, we considered systems with abundance of non-polar lipids. As it will be demonstrated, this approach allowed us to obtain a relatively stable multi-layered lipid system which can be considered as a plausible model of the tear film.

### Force field, general system setup, simulation parameters

Molecular dynamics simulations of the tear film model were carried out employing the coarse grain MARTINI force field which enables a near-atomistic description of molecular systems [44]. It was previously shown that this force field reliably reproduces phenomena occurring in various models of biologically relevant lipid systems such as lipid bilayers in cell membranes or lipid monolayers in the lung surfactant [45], [46]. MARTINI was also used in recent studies of the tear film with a good agreement between experiments and simulations achieved [42], [43], [47].

**Figure 1:**
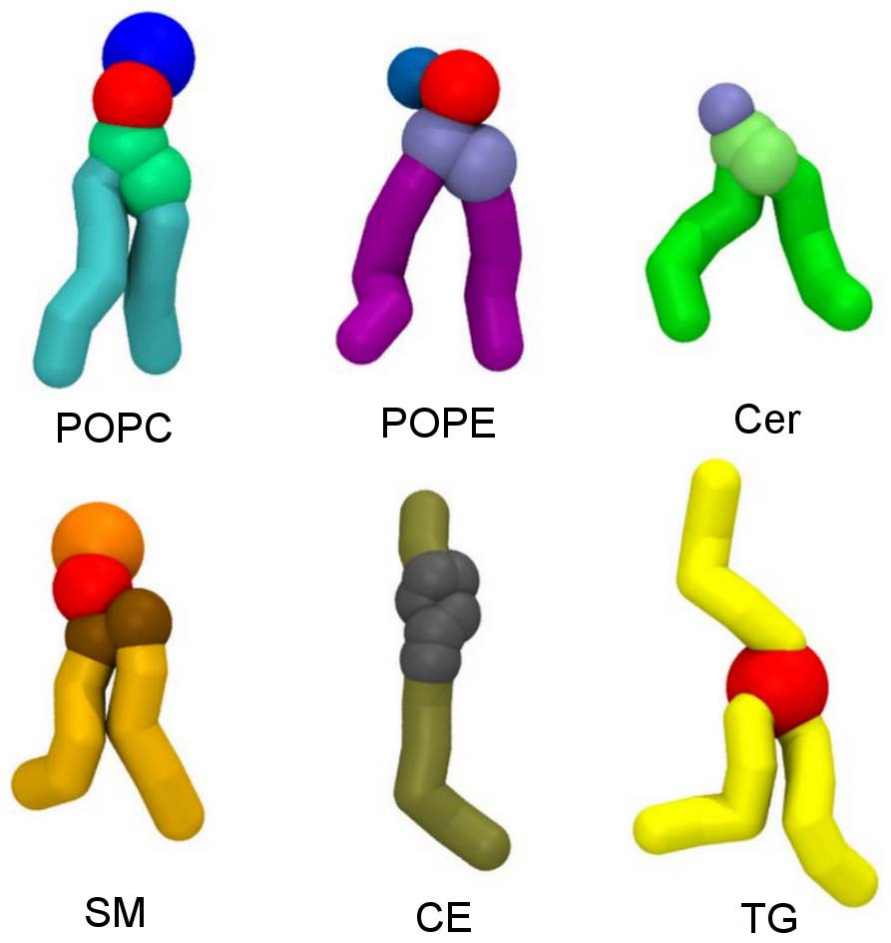
Coarse grain representations of lipid molecules considered in simulations.

The model of the tear film considered in our study consisted of a multicomponent lipid phase at the air-water interface. The lipid phase contained both polar (1-palmitoyl-2-oleoyl-phosphatidylcholine (POPC); 1-palmitoyl-2-oleoyl-phosphatidylethanolamine (POPE); N-palmitoyl-D-erythro-sphingosine (Cer); N-palmitoyl-D-erythro-sphingosyl-phosphorylcholine (SM)) and non-polar (glycerine trioleate (TG) and cholesteryl oleate (CE)) lipids. The coarse grain representation of these selected lipids is shown in Fig. 1. Original MARTINI parameters were used for all considered lipids with the exception of Cer, for which the force-field was not available and hence we employed the parameters of SM lipid with the headgroup bead exchanged to the P1 type. Simulations were performed in the periodic box with a slab of approximately 22,000 (87,000 and 347,000 in the case of the four-fold and sixteen-fold extended system respectively) water beads placed in the middle of the box. Note that each water bead in the MARTINI model corresponds to four water molecules. The box was elongated in one direction thus forming two independent air-water interfaces. Two symmetric lipid phases, each of the same composition, were placed at both interfaces (average data from both interfaces were used for density profiles analysis whereas one of the interfaces was selected for visualization of trajectories). Such a lipid arrangement which enables an approximately symmetric simulation system and improves the sampling is routinely used in simulations of lipid films [42], [45].

Simulations were performed employing the GROMACS 4.6.1 software suite [48]. A standard protocol for MARTINI-type simulations was employed [49]. Shortly, non-bonded interactions were cut-off at the distance of 1.2 nm employing the shift function from 0.9 nm. Short-range Coulomb interactions were cutoff at 1.2 nm with the shift from 0.0 nm. Equations of motion were integrated with the time-step of 20 fs. The temperature of 305 K was controlled independently for lipids and water phases employing the Berendsen thermostat algorithm with the relaxation time of 0.3 ps [50]. Most simulations were performed within the canonical ensemble, i.e., the size of the box was kept constant. In non-equilibrium simulations of lateral compression and decompression, lateral pressure was employed using semi-isotropic Parrinello-Rahman barostat with the relaxation time of 3 ps and compressibility of 3×10^−5^ [51]. Trajectories of 1200 ns were calculated in the case of constant volume simulations. The first 200 ns of each simulation were treated as equilibration and for that reason not taken into account in further analyses. In non-equilibrium simulations, the evolution of the system was typically followed for 500 ns.

### Simulated systems

The system was built starting from an equilibrated monolayer of POPCs in the amount of 200 molecules spread on each of two air-water interfaces. In order to create multicomponent lipid system, some of the POPC molecules were replaced with molecules of TG (9%), SM (5%), POPE (20%), and Cer (5%). The replacement was performed gradually, namely the simulation of at least 100 ns within the canonical ensemble was run after addition of each distinct lipid component. The slab of nonpolar lipids of the nonpolar thick layer was prepared and equilibrated in a separate simulation and then transferred in two copies to the polar lipid/air interfaces. The final system contained 244 POPC, 80 POPE, 20 Cer, 20 SM, 636 TG (from which 36 molecules comes from initially prepared monolayer), and 600 CE. The water subphase in the middle of the simulation box consisted of more than 20,000 water beads. It was further equilibrated for few tens of nanoseconds in the canonical ensemble, followed by a simulation with lateral pressure employed in order to generate snapshots with a desired level of lateral compression. These snapshots, upon further equilibration, were used as initial structures for production runs in canonical ensemble. Additionally, we performed non-equilibrium simulations of lateral compression and expansion in systems with either four-fold or sixteen-fold increased number of molecules; the latter having over 25,000 lipids in the simulation box.

## Results and Discussion

Simulations were performed at different lateral compressions to mimic varying surface pressure conditions expected during an eye blink [52]. To characterize the compression, we utilize the area per polar lipid (APPL) parameter defined as the lateral area of the simulation box per number of all polar lipids in one monolayer. First, equilibrium MD simulations, employing the canonical ensemble were performed so that the APPL was kept constant during each simulation. The values of APPL between 31.5 and 71 Å^2^ were applied in these simulations. As will be discussed in the following, two different types of behavior were observed with varying lateral compression. For low lateral compressions characterized by APPL > 60 Å^2^, the water-lipid interface was approximately planar, while for more compressed systems, at APPL ≤ 60 Å^2^, undulations of the lipid-water interface were observed. Additionally, non-equilibrium simulations were performed in which the system was laterally compressed either beyond or just before the collapse point of the lipid film and then laterally decompressed. In those simulations buckling and interface undulations occurred followed by a reorganization of the lipid film and formation of various three-dimensional structures.

In the following, first we discuss typical results obtained under equilibrium conditions in each of the two regimes, namely at APPL =  68 Å^2^ representing a laterally relaxed system, and APPL =  45 Å^2^ which corresponds to a relatively compressed interface. Then we present the results obtained during non-equilibrium simulations of lateral compression and decompression where strong reorganization of the lipid film occurs.

### Laterally relaxed lipid films

**Figure 2:**
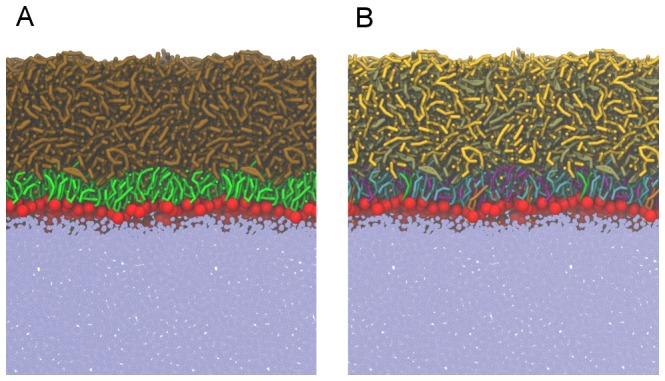
Typical snapshots of the laterally relaxed system (APPL = 68 Å^2^). An overall side-view with water beads shown in ice-blue, non-polar lipids in brown, headgroups of polar lipids in red, and tails of polar lipids in green (A); a detailed view of individual lipid components: TG in gold, CE in tan, POPC tails in cyan, POPE tails in purple, SM tails in orange, Cer tails in green, and all polar headgroups in red (B). For presentation purposes, in each snapshot two periodic images in the lateral direction are depicted (corresponding to about 22 nm).

**Figure 3:**
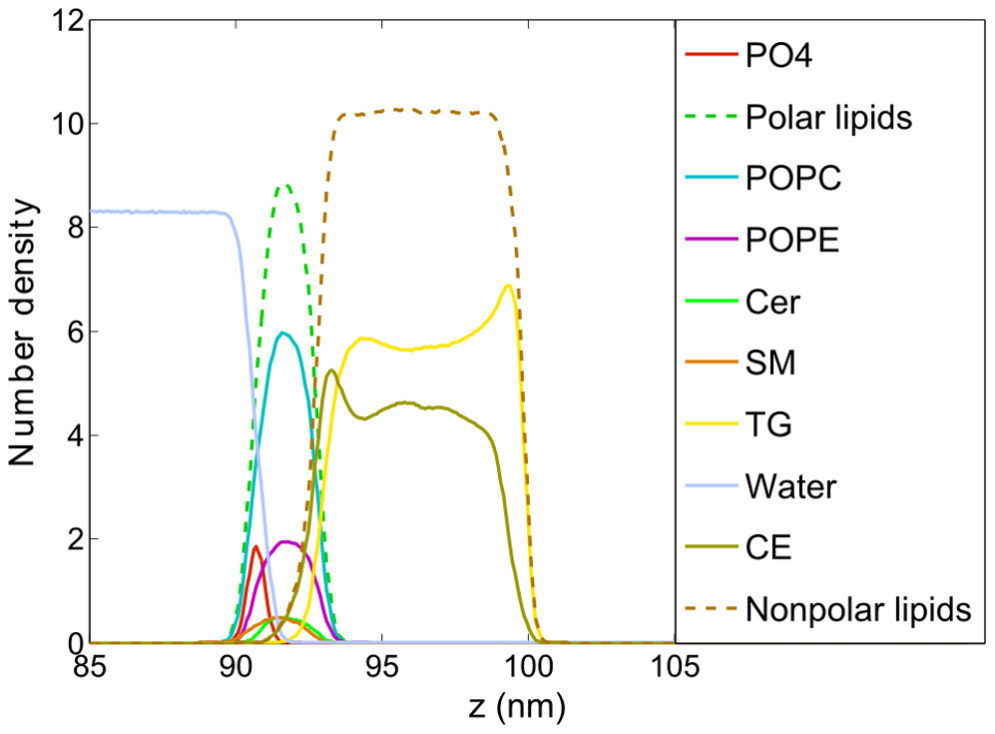
Density profiles of the system components at APPL = 68 Å^2^. The data are averaged over the simulation time from 0.2 μs to 1.2 μs.

In Fig. 2, a typical snapshot of the equilibrated system simulated at APPL =  68 Å^2^ is presented. Fig. 2A shows a general view of polar and non-polar lipids while Fig. 2B unravels a detailed picture of considered lipid composition. The lipid molecules are located at the water/air boundary, thus forming two interfaces: water/lipid and lipid/air. An ordered layered structure of the lipid film is visible. Polar lipids are located exclusively at the water/lipid boundary forming a thin monomolecular layer whereas the non-polar lipids form a relatively thick layer located atop the polar film. In the polar lipids subphase, further organization occurs, as lipid headgroups (shown in red) are oriented toward water while non-polar tails (shown in green) point in the direction of the non-polar layer. A significant overlap between polar lipid headgroups and water is present, demonstrating that the headgroup region is well hydrated while the tail region is dehydrated (see also Fig. 3). This behavior resembles the water/lipid boundary of both phospholipid monolayers at the water surfaces and hydrated phospholipid bilayers. The observed localization of polar lipids at the water/lipid interface is in agreement with previous MD studies of the tear fluid [42], [43], [47]. Localization of individual system components can be further elucidated based on the density profiles presented in Fig. 3. Separation between polar (dashed green line) and non-polar (dashed brown line) lipids is evident. Polar lipids effectively keep their non-polar counterparts apart from the water subphase. The polar lipid layer spreads for approximately 3.5 nm which approximately correspond to the length of POPC and POPE molecules. Polar headgroups of both POPC (in red) and POPE (in purple) are residing at the water-lipid boundary whereas the tails are pointing toward the non-polar lipid subphase. No substantial differences between localization of POPC and POPE lipids can be observed (see Fig. 2B) except of a somewhat deeper penetration of POPC headgroups toward the water phase caused by a larger size of PC headgroup with regard to the PE one (see Fig. S1 in the Supporting Information). Other polar lipid components, Cer and SM, are well mixed within the monolayer. SM lipids are shifted toward the water phase with regard to Cer, most likely due to the difference in their headgroups size, similarly to the difference observed between POPC and POPE. No lateral separation between polar lipids was observed.

Non-polar TG and CE molecules form an approximately 8 nm-thick subphase localized atop the polar lipid layer. The non-polar region is dehydrated; consequently, three well defined boundaries can be identified in the system concerning water/polar lipids, polar/non-polar lipids, and non-polar lipids/air. Although no substantial inhomogeneities in the non-polar layer are observed in simulation snapshots (see Fig. 2B), a detailed analysis of density profiles shown in Fig. 3 reveals some regularities in non-polar lipids orientation. Namely, at the polar/non-polar lipid interface, CE molecules penetrate closer toward polar lipids than TG, as the density of TG in the region of 91–93 nm is depleted with regard to that of CE. Carbon tails as well as the rings of CE molecules penetrate in-between the tails of polar lipids, whereas the long carbon tails of TG favor the orientation outward the polar layer (see the partial density profiles of CE atoms in Fig. S2 in the Supporting Information). The peak at the CE density profile at ∼93 nm results from the preference of CE toward polar lipids whereas the peak at the TG density profile at about 94 nm arises due to the compensation of the depleted CE molecules by TG. Non-polar lipids, taken all together, form a smooth interface with their polar counterparts (see the dashed ochre and green lines in Fig. 3). CE molecules in the bulk of the non-polar layer do not exhibit any orientation preferences and they mix homogeneously with TG molecules. At the non-polar lipid/air interface, the density of CE is depleted with regard to that of TG (Figs. 2 and 3) which is opposite to the behavior observed at the polar/non-polar lipids boundary.

TG molecules do not penetrate as close to the polar lipids region as the molecules of CE. Nevertheless, there is a non-negligible overlap between the density profiles of TG and polar lipids (see Fig. 3). Analysis of the density profiles of individual TG carbon tails (results not shown here) reveals that in the proximity of polar lipids, on average two TG tails interdigitate with the tails of polar lipids, while one TG tail points toward the bulk of the non-polar subphase. Thus, TG molecules attain a fork-like arrangement at the polar/non-polar lipids boundary. The density of TG molecules is elevated at the lipid/air boundary thus demonstrating that TGs predominantly occupy the surface of the lipid film. Also, an orientational preference of the TG lipid tails arrangement is observed, with TG tails pointing predominantly toward the air phase (see Fig. S2 in the Supporting Information). In can be concluded that polar lipids are significantly aligned in the polar layer at the water/lipid interface and, to some degree, lipid chains in the non-polar phase are aligned at both interfaces (polar/non-polar and lipid/air). Lipid chains in the bulk of the non-polar phase are disoriented.

The thickness of the non-polar lipid subphase at a given APPL (i.e., lateral compression) depends on the amount of non-polar lipids with respect to their polar counterparts. As demonstrated in previous computational studies, if there are not enough non-polar lipids present in the system under low lateral compression, these lipids cannot form a non-polar layer that would fully cover the polar lipid phase [42], [43]. In such a case, non-polar lipids form droplets that reside at the surface of the polar monolayer. In our simulations, the amount of non-polar lipids is relatively high, and thus a multimolecular non-polar coating is formed at the top of the polar lipid monolayer. Note that the presence of the non-polar coating is required to mimic the structure of the lipid film at the corneal surface. In present simulations, formation of the non-polar layer fully covering the lipid film was assured in all simulated systems even though the thickness of the non-polar layer varied with the lateral box size.

The following general conclusions can be made based on aforementioned results. In the laterally relaxed systems with the lipid composition mimicking the lipid composition of the tear fluid, a planar but multi-layer lipid film is created at the water surface upon system equilibration. Polar lipids form a monolayer at the water/lipid boundary. Their headgroups point toward the water phase and are relatively well hydrated, while their hydrocarbon tails are oriented in the direction of the lipid phase and dehydrated. The arrangement of different lipids in the polar monolayer is governed by their headgroup size, and no lateral inhomogeneities in the monolayer composition are observed. Polar lipids constitute a platform for non-polar lipid molecules, the latter forming a thick, dehydrated multimolecular layer located between the polar lipids and the air phase. In the non-polar lipid phase, CE molecules are enhanced at the non-polar/polar boundary and migrate in-between the tails of polar lipids. TG molecules are somewhat depleted in the vicinity of the polar phase. In contrast, they predominantly cover the lipid/air interface while the CE molecules are depleted there. Regarding stability, formation of the multilayer structure described here requires equilibration of the system at which the multilayer lipid arrangement is conserved on the microsecond simulation timescale. Then, it is very likely that such a state can persist at least on the timescale of seconds.

### Laterally compressed lipid films

**Figure 4:**
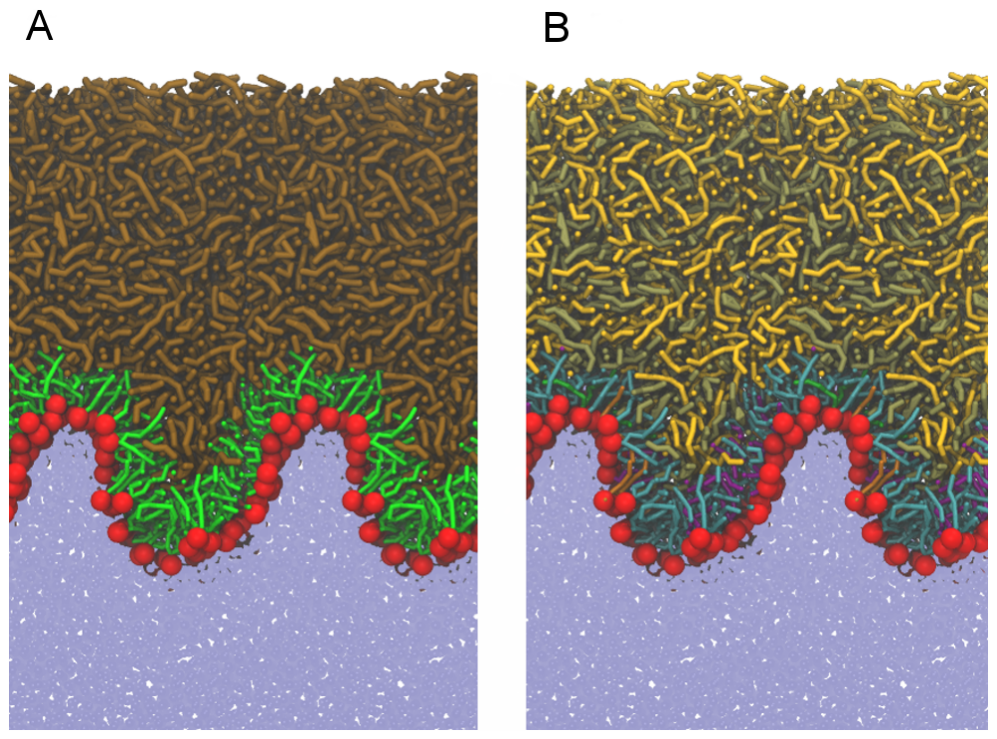
A typical snapshot of the laterally compressed system (APPL = 45 Å^2^). An overall side-view (A); a detailed view of individual lipid components (B). For color coding, see the legend of Fig. 2. For presentation purposes, in each snapshot two periodic images in the lateral direction are depicted (corresponding to about 18 nm).

A typical snapshot of the equilibrated system obtained in simulations at APPL = 45 Å^2^ is shown in Fig. 4A. The lateral compression causes undulations of the water/lipid interface, which spreads over the whole length of the simulation box. The integrity of the lipid film is preserved on the simulation timescale, as no rupture of the lipid layer or significant increase of water penetration across the lipid phase is observed. Single grains escape from aqueous to air phase but this process is comparable in both relaxed and laterally compressed systems. Note, however, that MARTINI force-field cannot properly predict water penetration because large beads (each corresponding to four real water molecules) are used for modelling the water phase.

Polar lipids follow the undulations orienting their headgroups locally toward the water phase. Similar behavior of the lipid film was observed in previous MD studies [45]. Some of non-polar lipid molecules fill the valley-like structures; however, most of non-polar lipids reside at the top of the undulated water/lipid interface. Overall, the role of both lipid types is the same as in the case of the laterally relaxed conditions, i.e., polar molecules reside at the water/lipid boundary separating the non-polar layer from water whereas non-polar lipids form a thick coating covering the polar sub-layer. Interestingly, the lipid/air interface formed by non-polar lipids is, on average, smooth. Hence, on the simulation time- and length-scales, undulations of the water/lipid interface do not manifest at the lipid/air interface. This effect requires the presence of an abundance of non-polar lipids with regard to the polar ones, as similar behavior was not observed in previous studies where non-polar to polar lipids ratio was relatively low [42], [43]. It can be postulated that a thick non-polar lipid layer exhibits a protective effect on the tear film keeping the air/lipid interface locally smooth. This effect is likely to be enhanced under physiological conditions, as the thickness of the non-polar lipid layer present in real tear films is typically several times larger than that employed here [40].

**Figure 5:**
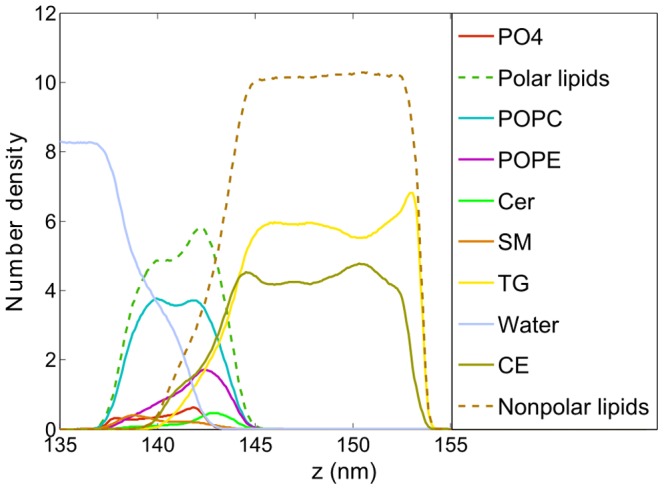
Density profiles of individual system components at APPL = 45 Å^2^ under equilibrium. The data are averaged over the simulation time from 0.2 μs to 1.2 μs.

**Figure 6:**
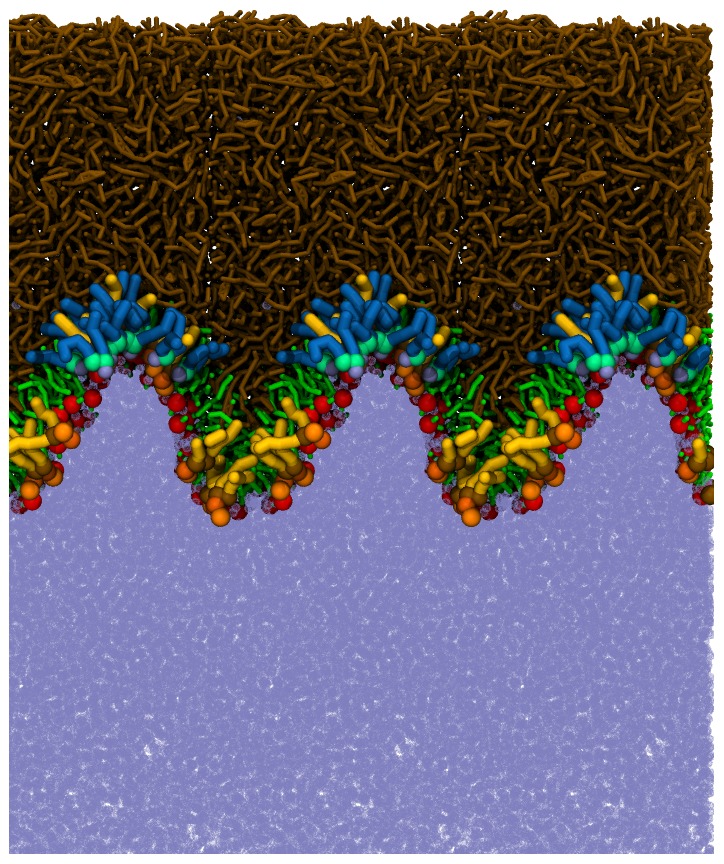
The sorting of polar lipids in regions of different curvature in the compressed system (APPL = 45 Å^2^). Color coding: SM tails in gold and headgroups in orange, Cer tails in blue and headgroups in light-green; POPC and POPE tails in green and headgroups in red, non-polar lipids (TG and CE) in brown, water beads in ice-blue. For presentation purposes, three periodic images in the lateral direction are depicted (corresponding to about 27 nm).

The density profiles of selected system components are shown in Fig. 5. Separation of non-polar lipids from the water phase by polar lipids is evident; a somewhat larger overlap between the non-polar lipids and water density profiles is due to the presence of undulations and does not correspond to local penetration of water into the non-polar phase. Undulations of the water/lipid boundary are also reflected in the bimodal distribution of polar lipids. In the polar sub-layer, a separation between different lipid types occurs. The density profiles in Fig. 5 reveal that POPE molecules preferentially occupy the hill-like positions at the undulated water/lipid interface (the POPE distribution curve peaks close to the polar subphase) whereas POPC lipids are distributed almost homogeneously. This particular sorting of lipids can be rationalized by different sizes of POPE and POPC headgroups, as the small heads of POPE are known to prefer the regions of negative curvature while those of POPC prefer positive curvature positions [53]. A sorting is observed also in the case of SM and Cer molecules. Namely, as evident in the density profiles shown in Fig. 5 and the snapshot presented in Fig. 6, ceramide molecules prefer to be located in the hill-like positions close to the non-polar phase. That is, they prefer negative curvature regions, whereas SM lipids localize in the valleys close to the water phase. This separation is driven by the difference in the headgroup size and spontaneous lipid curvature, as in the case of POPE and POPC [53]–[55].

In the laterally compressed system, the non-polar lipids behave similar as in the relaxed one. Namely, CE localizes preferentially close to the polar layer with its chains interdigitating with the chains of polar lipids. TG is also present close to the polar layer, however, it is depleted there with regard to that of CE. Similar differences between TG and CE have also been noted by Vuorela et al. [56]. The orientation of non-polar lipid is governed by water/lipid interface undulations and follows that of their polar counterparts. A small protuberance observed in the CE density profile in Fig.5 at approximately 140 nm stems from an enhanced tendency of CE to reside in valley-like structures with regard to TG. At the lipid/air interface, the TG density is increased with respect to that of CE (see Fig. 5) demonstrating that this interface is effectively covered by the tails of TG molecules, as was the case in the laterally relaxed system. The lipid/air interface is locally flat, thus the already mentioned protective effect of non-polar lipids at the lipid/air interface is observed also here.

### Non-equilibrium squeezing of the lipid film

In order to study the lipid film under conditions of lateral squeezing and to account for eye blinks, we performed non-equilibrium MD simulations applying lateral pressure. The goal here was to investigate the dynamics during the lateral squeezing and, if possible, the behavior of the system beyond the lipid film collapse point. Similar conditions are expected during eye blink when the tear film undergoes relatively rapid compression and decompression events. The water/lipid interface, as discussed beforehand, is noticeably undulated at APPL ≤ 60 Å^2^. A typical wavelength of undulations formed under these conditions is comparable with the lateral size of the simulation box (see Fig. 4A) and hence finite-size artifacts may be expected upon further squeezing [57]. To avoid such effects, the systems with either four- or sixteen-fold more lipids were employed for non-equilibrium squeezing simulations. The value of lateral pressure was chosen to result in the compression rate that approximately corresponds to the rate of the tear film squeezing during a typical eye blink. We assumed a human eye diameter equal to 25 mm, and the time of an eyelid down- and up-movement in a spontaneous blink to be approximately 100 ms and 250 ms, respectively [58]. Hence, the average rate of the tear film compression and decompression can be estimated at about 0.1 m/s and 0.04 m/s, respectively.

**Figure 7:**
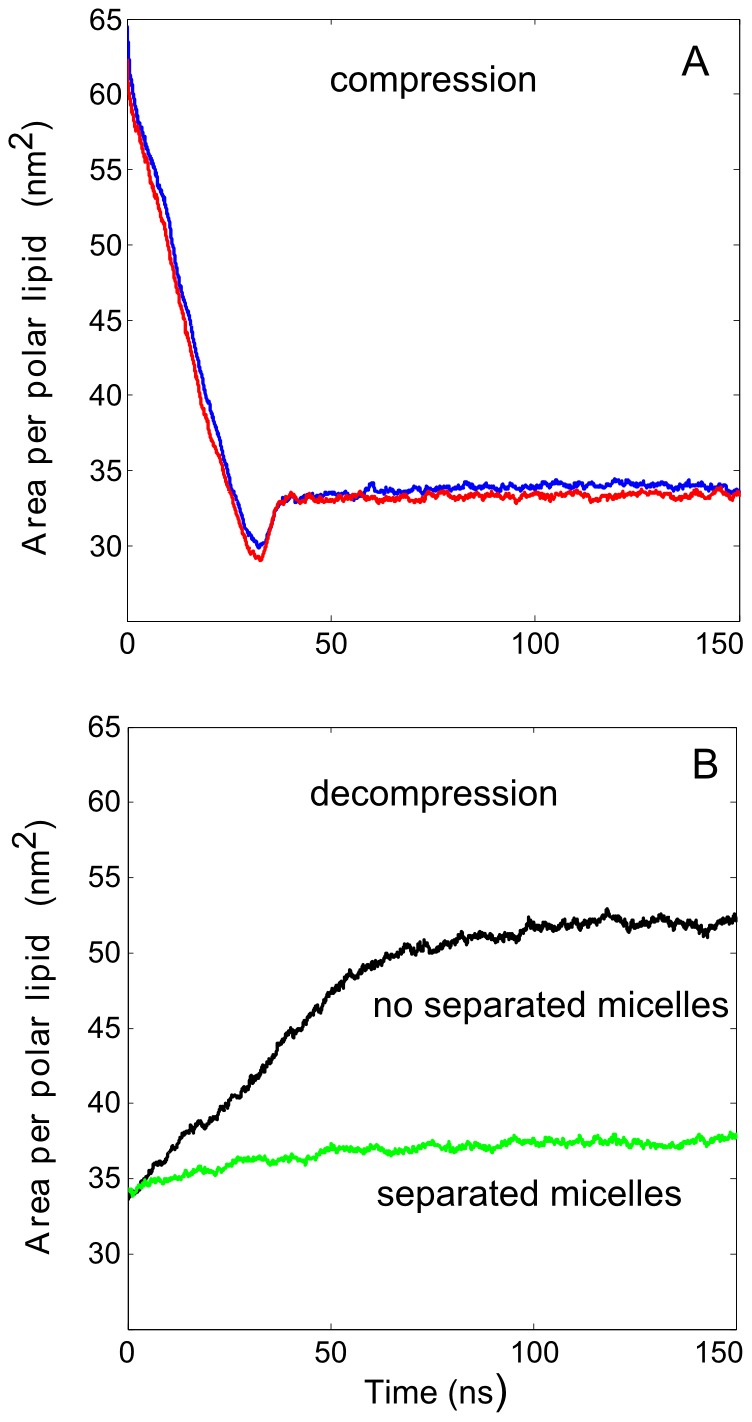
The area per polar lipid as a function of simulation time in non-equilibrium simulations of lateral compression. The lateral pressure of 1 bar was used, each curve represents an independent MD trajectory (A); and decompression under the lateral pressure of at −6 bars (B) with the decompression simulation initiated either prior (black curve) or after (green curve) the inversed micelles separated from the polar monolayer in a previous simulation of lateral compression.

In Fig. 7A, the APPL is shown as a function of simulation time during the lateral squeezing (lateral pressure of 1 bar) of the four-fold extended system. The results obtained in two independent MD trajectories are presented. In the initial stage of both trajectories (<35 ns), the response of the system to the lateral compression was approximately linear, with APPL decreasing in time until it was reduced to approximately 30 Å^2^. Note that the linear compression rate in simulations was approximately equal to 0.2 m/s, thus comparable to that experienced by the tear film during the closing of an eyelid in a typical eye blink. During this phase, undulations of the water/lipid interface, similar to those described beforehand for the system with APPL = 45 Å^2^, were observed and the system behavior was qualitatively the same as detailed previously under equilibrium conditions (i.e., the interface resembled that depicted in Fig. 4). With further lateral compression, a remodeling of the polar lipids layer occurred. Namely, irregular bulging of the monolayer appeared and separate aggregates of polar lipids in a shape of elongated cylinders and droplets attached to the folded monolayer were formed.

**Figure 8:**
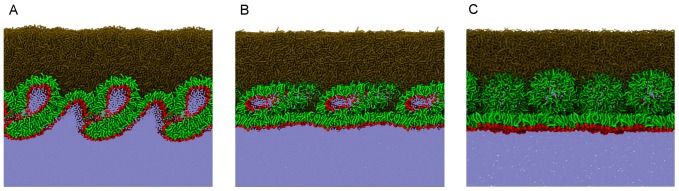
Typical simulation snapshots taken during the non-equilibrium lateral compression. The snapshots are taken before (A) and after (B) extrusion of polar lipids and water to the non-polar subphase; and during decompression (C). For color coding, see Fig.2A. For presentation purposes, in each snapshot three periodic images in the lateral direction are depicted (corresponding to 47–50 nm).

**Figure 9:**
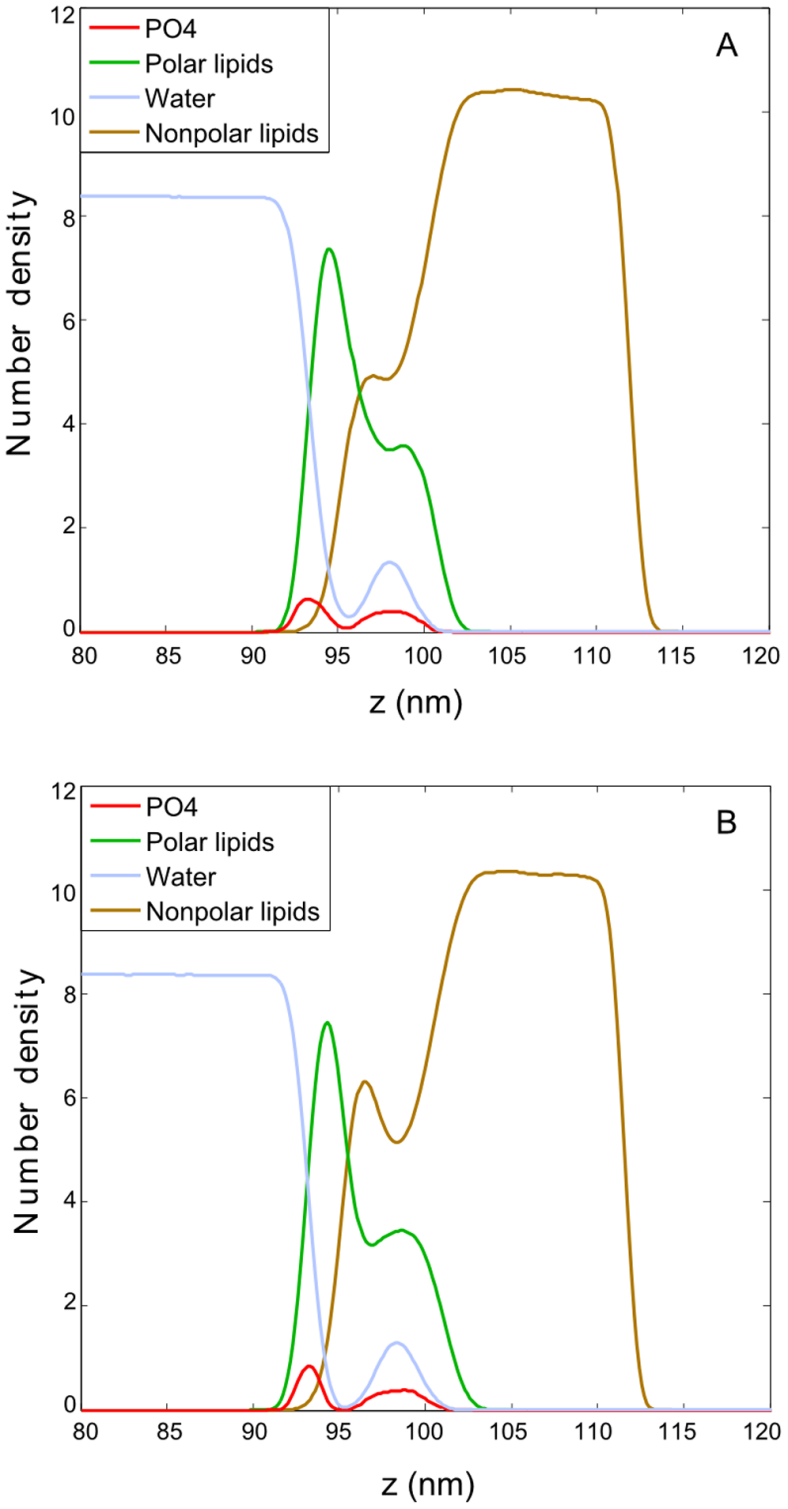
Density profiles calculated during the non-equilibrium lateral compression. The profiles are taken in the initial (40-100 ns) (A) and final (100-150 ns) (B) phase of the non-equilibrium lateral squeezing.

**Figure 10:**
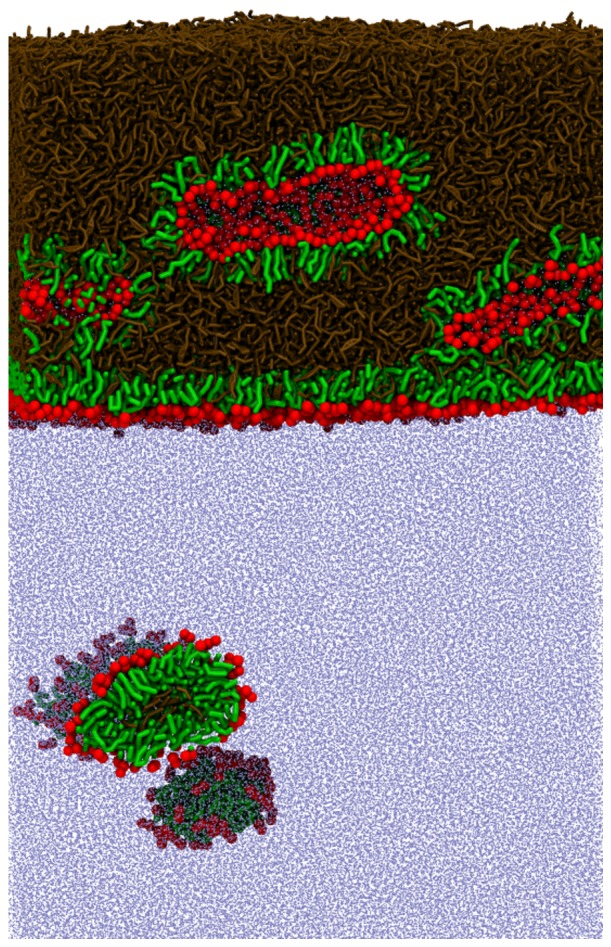
Simulation snapshot of the extended, laterally compressed system. The snapshot is taken at about 400-equilibrium compression trajectory of the sixteen-fold extended system. Inversed micelles are present in the non-polar lipid phase (both attached to and detached from the polar lipid layer. A micelle is present in the water phase. For color coding, see Fig.2A. Note that the picture represents a single simulation box, i.e., no lateral periodicity was applied. The lateral size of the box is of about 43 nm.

A typical simulation snapshot taken at this stage is depicted in Fig. 8A where the three-dimensional structures formed consist of clusters of water enclosed by polar lipid molecules. This is further evidenced in the density profiles shown in Fig. 9A which were calculated for one of the compression trajectories. The profiles in the range between 95 and 100 nm correspond approximately to the region of the tear film where the three-dimensional structures are formed. The protuberances observed in this region at density profiles of both polar lipids and water demonstrate that the observed three-dimensional structures consist of water and polar lipids. These structures undergo some reorganization during further compression (see Figs. 8B and 9B). Namely, they are remodeled into inverse micelles, with polar lipids encapsulating water clusters. Notably, the micelles do not completely detach from the polar lipid monolayer. The process of micelle formation leads to smoothening of the polar lipid monolayer and to suppression of interface undulations. On the simulation timescale, we did not observe migration of micelles toward either the bulk or the non-polar phase; they remained attached to the monolayer of polar lipids. Different types and behavior of three-dimensional structures were observed in simulations of compression of the sixteen-fold extended system (hence confirming the non-negligible role of finite size effects). In this extended system, formation of inversed micelles in the non-polar phase was observed and these micelles were able to detach from the polar/non-polar boundary (Fig. 10). Moreover, some of polar lipids were relocated to the water subphase where they formed micelles (Fig. 10).

During all lateral compression simulations, the lipid/air interface stayed relatively smooth, similarly to the previously described laterally compressed systems under equilibrium. Note that the lipid arrangement formed upon strong lipid film compression is qualitatively different than that in the three-layer system observed in equilibrium simulations. The new feature is that the polar sub-layer is not a flat monomolecular layer of polar lipid molecules, but it has a form of a monolayer covered by irregular bulges of attached inversed micelles formed by polar lipids and water.

To model the opening of eye lids during blinks, we performed simulations of lateral decompression of the previously compressed lipid films with two types of initial configurations employed. First, the partially compressed film was taken, with three-dimensional structures formed but no micelles present (similar to that depicted in Fig. 8A). Second, the film with fully formed inversed micelles was used (similar to that depicted in Fig. 8B). The lateral pressure of −6 bars was employed. In Fig. 7, APPL is shown as a function of time for simulations with both types of initial configurations. During lateral decompression of the system starting without micelles, APPL increases significantly (initial lateral decompression rate equal to 0.09 m/s) due to partial re-incorporation of polar lipids from the three-dimensional structures back to the polar monolayer. This fusion process is not complete because not all structures merge with the monolayer on the simulation timescale, as evidenced by saturation of APPL after 15 ns and the final value of APPL achieving lower level than that observed in the initial system before the squeezing. In the case of lateral decompression initiated with the fully formed micelles, no significant re-incorporation of the squeezed-out material into the monolayer was observed; the rise of APPL with simulation time is very moderate (initial lateral decompression rate equal to 0.06 m/s) and saturates at about 8 ns. In this simulation, however, some remodeling of the micelles was observed, as evidenced in Fig. 8C.

## Conclusions

The main aim was to study at the molecular level biophysical properties of a realistic tear film model under conditions mimicking those experienced by the tear film under physiological conditions. Molecular-level organization of lipid tear film was studied employing coarse grain molecular dynamics simulations. The lipid composition used here directly reflects the lipidome of the aqueous tear fluid [32]. This lipidome is still of qualitative nature and that further studies are required to arrive at a fully quantitative result. Our model also includes a relatively large amount of non-polar lipids, simulating those of the Meibomian glad origin, which are experimentally known to be the dominant component of the lipid tear film and which are shown here to have a crucial role in modulating lipid film behavior. In real eye, such a clear distinction between aqueous tear lipids and meibomian glad lipids may not necessarily be the case. As pointed out in the recent study by Borchman *et al.*, the meibum may also be a source of tear lipids [38]. It is also worth noting that lipid-protein interactions are supposed to play an important role in the tear film. However, this issue was beyond the scope of the current study.

Regarding the role of individual lipid components, our simulations demonstrate that polar phospholipids separate non-polar lipids from the water phase, constituting a monomolecular uniform platform at which a thick non-polar lipid layer is formed. Polar lipids organize in such a way that their hydrophilic head groups interact with the water phase whereas hydrophobic tails orient toward non-polar lipids. Non-polar lipids are in contact with the tails of their polar counterparts. Such a lipid arrangement was found to be stable upon lateral compression. These results are in agreement with recent findings where MD simulations of lipid tear film were employed [42], [43].

We evaluated the role of non-polar lipids in the tear film; this was possible due to the inclusion of the dominant thick non-polar lipid layer in the present simulations. The non-polar lipid layer is organized; lipids orientation and composition are homogenous in the bulk of the non-polar layer whereas at both polar/non-polar and lipid/air interfaces preferential molecular orientations and lipid composition can be identified. Namely, CE molecules are dominant at the polar/non-polar interface with their short carbon tails and rings penetrating in-between the chains of polar lipids and long tails preferentially pointing toward the non-polar phase. TG molecules are somewhat depleted at the polar/non-polar boundary with, on average, two TG tails interdigitating with the tails of polar lipids. On the other hand, at the lipid/air interface, CE molecules are strongly depleted, and hence the tear lipid surface is predominantly covered by triglycerides. TG molecules at the lipid/air boundary exhibit orientational preference, with their tails pointing toward the air. Thus, we predict that sorting of non-polar lipids occurs at both polar/non-polar and lipid/air boundaries.

It should be noted that pure CE undergoes a phase transition to a smectic phase at ∼314 K [59]. We performed test simulations with bulk CE around 314 K and did not observe such transition. Hence, the MARTINI model of CE employed here does not reproduce the smectic transition in the bulk of pure CE. However, it was shown experimentally that an addition of TG to CE abolishes all liquid-crystalline transitions in the TG+CE mixture [60]. Similar effect was experimentally demonstrated also in more complicated lipid mixtures (TG+CE+cholesterol) [61]. Therefore, as the non-polar phase considered in our model is a mixture of CE with TG, it should not exhibit phase transitions at the temperature 310 K employed in simulations. There are recent experimental reports suggesting a gel-like state of non-polar lipids in the tear film [62]. We do not see such effects in our simulations. However, we cannot exclude formation of gel-like aggregates occurring at longer time- and length-scales than these explored in our simulations. It is worth to note another limitation of MARTINI model related to water penetration. Namely, the role of non-polar lipids in preventing water evaporation cannot be studied employing MARINI force field due to the coarse-grain nature of MARTINI water grains.

The lipid tear film undergoes constant changes during eye blinks due to the action of eye lids. At the molecular level, this action can be modeled as alterations of the lateral pressure of the film. Under moderate lateral pressures (APPL > 60 Å^2^), the lipid film remains flat on a nanometer length scale. However, when laterally compressed (APPL < 60 Å^2^), we observed a significant restructuring of the film. First, sorting of polar lipids occurs under moderate lateral compressions. The water/lipid interface undulates, and lipids arrange depending on their head group size. POPE and Cer prefer the regions with a negative curvature, SM preferentially locates in the positive curvature regions, while no preference for POPC is observed. Thus, upon lateral compression, polar lipids adjust their positions to the varying shape of the water/lipid interface. Such a sorting of polar lipids may play roles in physiology and biomechanics of the tear film. A second notable effect observed in simulations upon lateral compression of the tear film is a relatively flat shape of the lipid/air interface. Undulations of the water/lipid interface are not propagated to the surface of the tear film on the time- and length scales of simulations. Hence, the thick layer of non-polar lipids plays a stabilizing role for the tear film surface during lateral compression. This adds to the previously described protective role of the non-polar lipid layer in preventing water evaporation, as well as to its role for optical properties of the tear film [2], [22].

The most significant changes in the structure of the lipid tear film were observed in the non-equilibrium lateral compression/decompression simulations. Namely, we observed undulations of the water/lipid boundary followed by transfer of some of polar lipids toward both non-polar and water phases. This resulted in formation of inverse micelles in the non-polar lipid layer with water encapsulated by polar lipids, as well as micelles in the water phase. Some of these micelles were attached to the polar/non-polar lipid boundary.

The action of eye lids during blinks introduces significant variations of the lateral pressure encountered by the lipid tear film layer. Note that the time needed for equilibration of phospholipid monolayers in Langmuir through experiments is in the range of at least minutes [47]. Hence, it can be assumed that the tear film under physiological condition is far from the thermodynamic equilibrium as the average time interval between individual eye blinks are in the range of about 6 seconds [63]. This gives a basis to assume that the tear film structure obtained in non-equilibrium lateral compression and decompression simulations resembles organization of the real tear film much closer than that obtained in the equilibrated systems.

The following molecular-level picture regarding the structure of the lipid tear film can be drawn based on the present model. The lipid tear film is not a locally flat assembly with a monolayer of polar lipids at the water/lipid interface and a thick layer of polar lipids at the top. Instead, our results suggest that even on a nanometer length scale, the water/lipid interface is a dynamic and non-homogeneous region with polar lipid material and water forming three-dimensional structures at the polar/non-polar lipid boundary. These structures are kinetically stable on the simulated time scale (microseconds) and they form and reorganize due to the action of eye lids during blinks. Some of polar lipids are also transferred to the non-polar and water phases. Regarding the lipid/air interface, we showed that it remains locally flat as it seems to be well isolated from the water/lipid interface. Recent experimental reports point out that the surface of the eye is macroscopically irregular and not smooth [40], [64]. However, since length- and time scales of experiments are far beyond those probed in MD simulations, it is difficult to ascertain whether such irregularities are connected with the structures observed at the nanometer and microsecond scale in the present work.

We can speculate about a possible role of the three-dimensional structures adsorbed at the water/lipid boundary or residing in its vicinity. The transfer of polar lipids from the monolayer to the inversed micelles and micelles upon lateral compression allows for stabilization of the tear film during eye blinks, as polar lipids can be transferred out of the polar monolayer when the area of the film decreases. These three-dimensional assemblies can be viewed as a reservoir of polar lipids which can be transferred back to the polar monolayer if required. This may point to a possible role of these structures in some of pathological conditions connected with the decreased stability of the tear film.

## Supporting Information

Figure S1 **Density profiles of polar lipids at APPL = 68 Å^2^.** The data are averaged over the simulation time from 0.2 μs to 1.2 μs. Profiles of headgroups and both sn-1 and sn-2 chains (‘tail B’ and ‘tail A’, correspondingly) of POPE and POPC lipids are depicted.
(TIFF)

Figure S2 **Density profiles of non-polar lipids at APPL = 68 Å^2^.** The data are averaged over the simulation time from 0.2 μs to 1.2 μs. Density profiles of both glycerine backbone and tails are shown for TG. In the case of CE, profiles of both ring system and tail are depicted.
(TIFF)
