# Supplementary figures and images for: Organization of Lipids in the Tear Film: A Molecular-Level View
Source: PLoS One. 2014 Mar 20;9(3):e92461. doi: 10.1371/journal.pone.0092461 (PMC3961367; doi:10.1371/journal.pone.0092461)

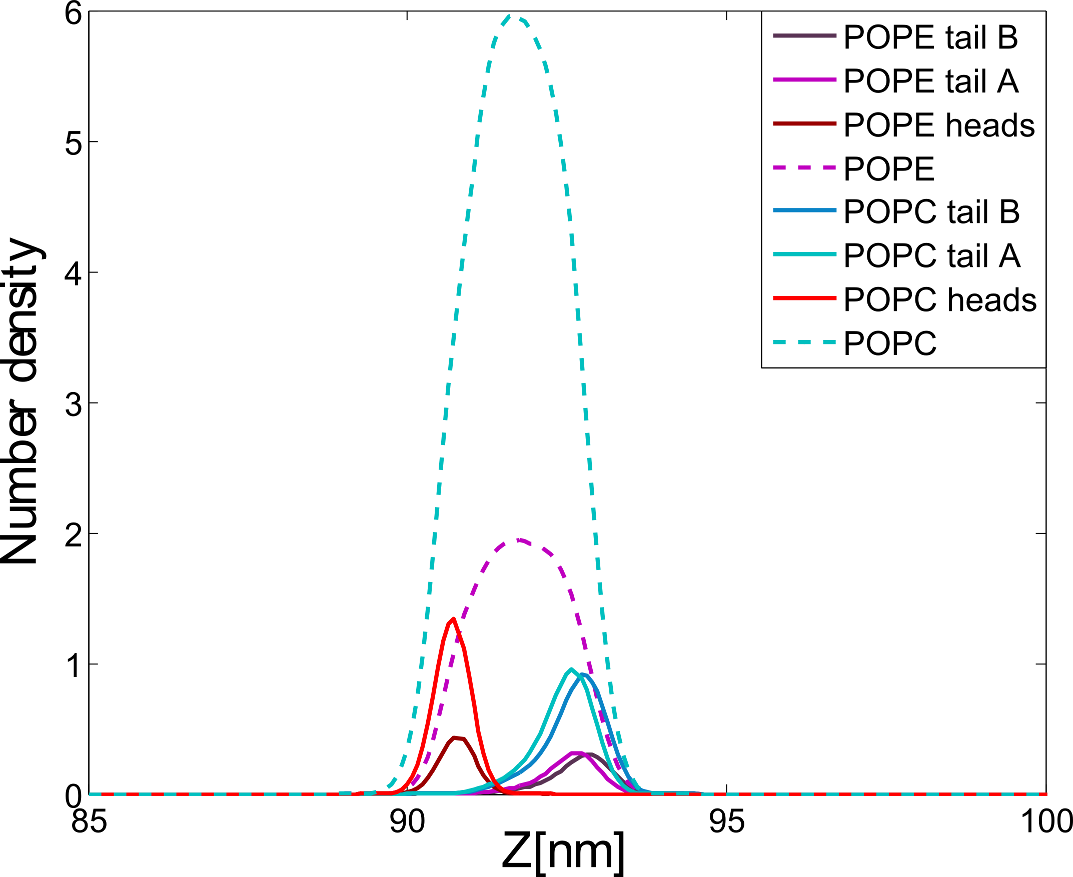

Supplement: Figure S1 — Density profiles of polar lipids at APPL = 68 Å2. The data are averaged over the simulation time from 0.2 μs to 1.2 μs. Profiles of headgroups and both sn-1 and sn-2 chains (‘tail B’ and ‘tail A’, correspondingly) of POPE and POPC lipids are depicted. (TIFF) [file pone.0092461.s001.tif]

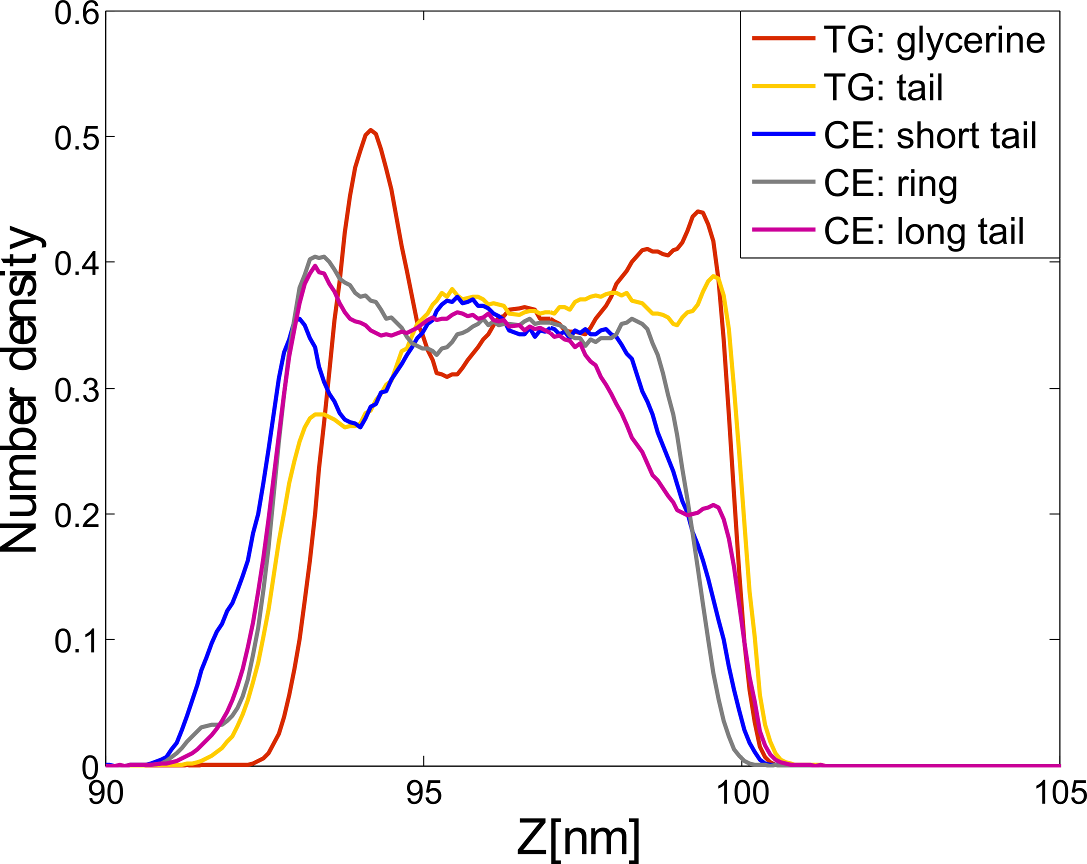

Supplement: Figure S2 — Density profiles of non-polar lipids at APPL = 68 Å2. The data are averaged over the simulation time from 0.2 μs to 1.2 μs. Density profiles of both glycerine backbone and tails are shown for TG. In the case of CE, profiles of both ring system and tail are depicted. (TIFF) [file pone.0092461.s002.tif]
